# Post operative abdominal wall mucormycosis infection after laparotomy for bowel perforation

**DOI:** 10.1016/j.idcr.2024.e01998

**Published:** 2024-05-24

**Authors:** Neha Kumta, Lawrence Huang, Gururaj Nagaraj, Lindsey Papacostas, Shradha Subedi

**Affiliations:** a Intensive Care Department, Sunshine Coast University Hospital, Australia; b Infectious Diseases Department, Sunshine Coast University Hospital, Australia; c Department of Microbiology, Sunshine Coast University Hospital, Australia; d University of Queensland, Australia

**Keywords:** Infectious diseases and parasitology, Pathology, Surgery, Intensive care

## Abstract

Mucormycosis is a devastating disease with a high mortality rate, typically affecting immunosuppressed individuals. Postoperative surgical site infections due to mucromycosis are rare, with only a handful of cases reported in the literature. Here, we describe a fatal case of post operative abdominal wound infection caused by mucormycosis in an immunocompetent man in his 70 s, who developed the infection following a laparotomy for bowel perforation. Initially, the growth of fungal species from a superficial wound swab was not considered significant until the patient exhibited signs of worsening sepsis. Limited operative debridement was performed for prognostication, in accordance with the family’s wishes. There was evidence of extensive significant invasive fungal infection, marked by necrosis extending into the abdominal wall fat and muscle. The patient was then transitioned to comfort measures and subsequently died. This case emphasizes the importance of maintaining a high level of clinical suspicion for mucormycosis, even in patients with minimal risk factors, and highlights the importance of prompt and aggressive treatment.

## Introduction

Mucormycosis refers to any infection caused by fungi within the Mucorales order. The infection is typically seen in immunosuppressed individuals and is characterized by infarction and necrosis of host tissues due to invasion of vasculature by fungal hyphae. Mucormycosis poses challenges in diagnosis, progresses rapidly, and has a substantial burden of morbidity and mortality [1].

Our case illustrates a rare and fatal case of post-operative mucormycosis abdominal wound infection in a patient following laparotomy for bowel perforation. The infection arose spontaneously in a host with a poor reserve, but without any traditional immunosuppressive risk factors. The growth of a fungal species from a superficial wound on day 9 was initially thought to be of unclear significance, until there was clinical evidence of abdominal wall wound infection on day 17. No breaches in infection control or other hospital-related environmental exposures were identified as the source of infection.

Though a rare cause of post-operative wound infections, clinicians should consider invasive fungal infection in patients with post operative wound infections that fail to respond to antibiotic therapy. Any growth of fungal species, including those from superficial swabs should be considered clinically significant and thoroughly investigated until proven otherwise. Timely and aggressive surgical intervention and systemic antifungal therapy is crucial for patients who are candidates for full active measures.

## Case presentation

A male in his 70 s with a history of chronic obstructive pulmonary disease and previously treated throat cancer in remission presented to a regional hospital with acute onset abdominal pain, in the context of intermittent abdominal discomfort for the past month. He had no history of diabetes mellitus and was not treated with chemotherapy or other immunosuppressive therapy in the preceding year.

Computerized Tomography (CT) of the abdomen demonstrated large volume pneumoperitoneum with likely faeculent peritonitis secondary to perforation of a hollow viscus of unclear underlying etiology. The patient was promptly commenced on broad-spectrum antibiotics and urgently underwent right hemicolectomy and washout. During surgery, extensive fecal contamination and a distal transverse colon perforation was identified, though the cause of this was unclear. His abdomen was temporarily closed using negative pressure wound therapy and he was admitted to the Intensive Care Unit (ICU) intubated and ventilated. The patient developed multiorgan failure with acute oliguric renal failure, requiring continuous renal replacement therapy.

Histopathology from the resection demonstrated no evidence of fungal hyphae within the bowel. He had a repeat laparotomy and wash out on Day 4 and then proceeded to a left hemi-colectomy on Day 6 which revealed a splenic flexure mass, later determined to be a poorly differentiated adenocarcinoma (state pT3 pN1b). Again, there was no evidence of fungal hyphae on histopathology specimens from this resection. A loop ilieostomy, away from the laparotomy wound was performed. The patient was extubated on day 11 and antibiotics were discontinued on day 14 after a surgical review of the laparotomy wound revealed no clinical signs of infection.

Unfortunately, the patient deteriorated on Day 16 and required re-intubation for respiratory failure. On Day 17, concerns rose about an underlying septic process, prompting the reinstatement of antibiotics. The abdominal wound underwent local debridement in the ICU, due to the isolation of fungal growth from a superficial wound swab taken on day 9. On day 1 of incubation of the abdominal wound tissue, there was fungal growth which had microscopic features consistent with a mucomycetes/order Mucorales species. Histology showed active inflammation with fungal spores with hyphae. The patient was commenced on intravenous liposomal amphotericin B (5 mg/kg). Given the patient's protracted clinical trajectory, the next of kin agreed to only a limited operative debridement to ascertain the extent of fungal infection for prognostic considerations. Repeat surgery on Day 19 showed significant invasive fungal infection, marked by necrosis extending into the abdominal wall fat and muscle.

The patient’s family expressed a strong preference against aggressive interventions, leading to a shift towards comfort-based approach. The patient deceased in ICU on Day 20. Since the infection occurred in a hospital setting in a surgical wound, a review of the infection control practices related to surgical care and wound management was undertaken. A thorough investigation found no breaches in standard infection control practices, especially concerning wound management. Additionally, a comprehensive review of fungal culture results from swabs and tissues of ICU and surgical ward patients over the preceding twelve months revealed no detection of mucor or other fungal species to suggest a nosocomial source of infection.

**Fig. 1:**
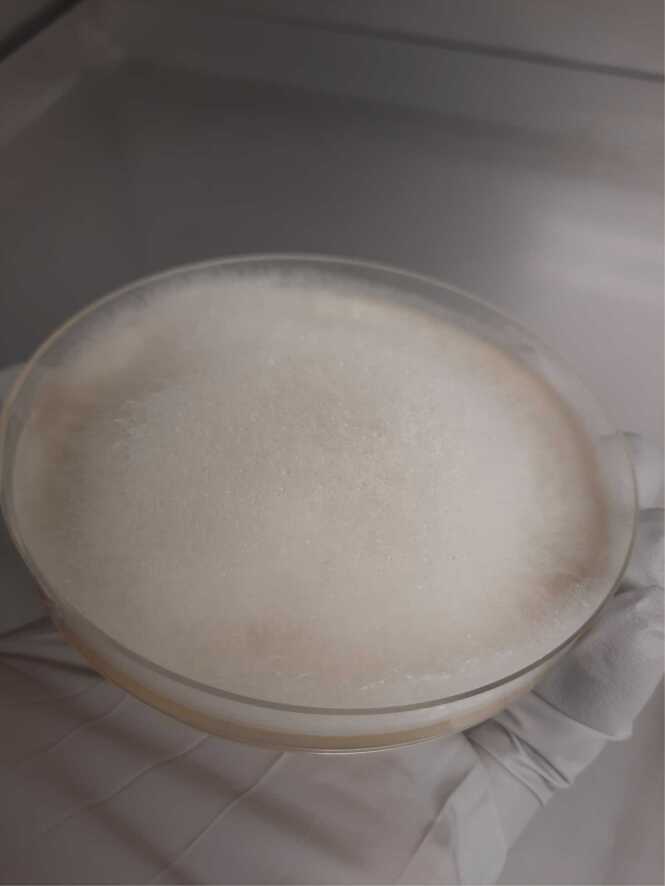
Agar plate demonstrating a cream fluffy fungus rapidly growing upwards in a classic “lid-lifter” formation.

**Fig. 2:**
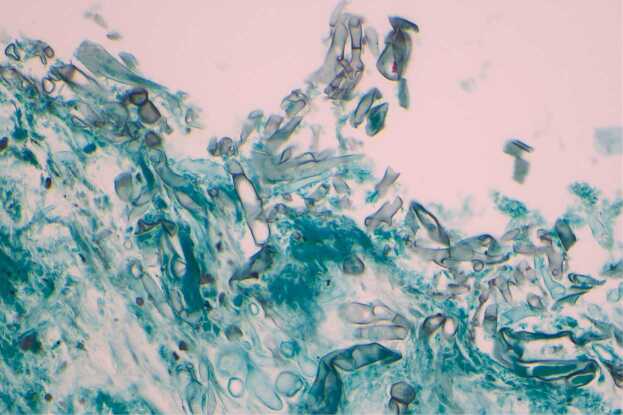
Histopathology smear demonstrating wide aseptate fungal hyphae, Grocott 40 ×.

**Fig. 3:**
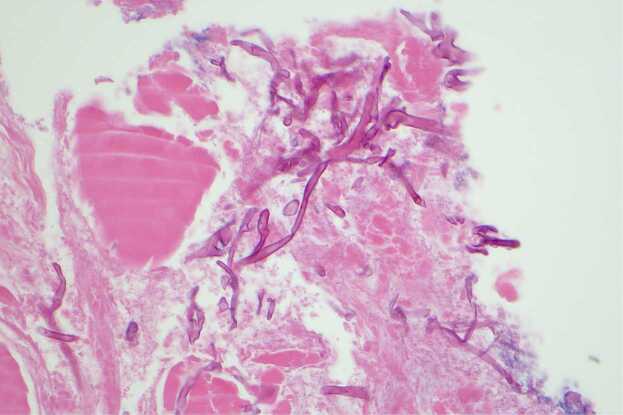
Histopathology smear demonstrating wide aseptate fungal hypahe, H&E 40 ×.

**Fig. 4:**
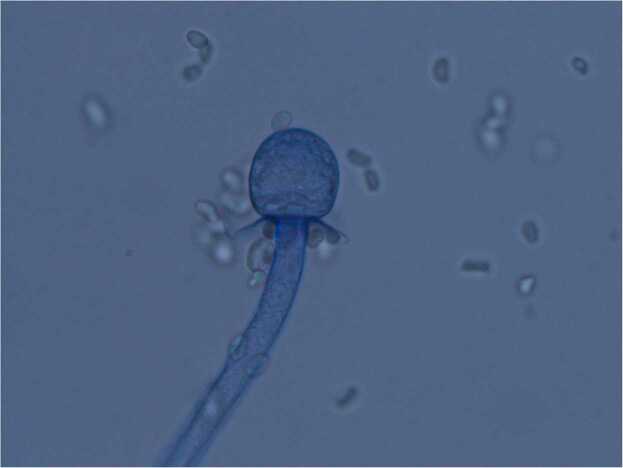
Microscopy demonstrating dark sporangia (1) and wide ribbon like non-septate hyphae (2). No rhizoids seen at the junction of the sporangiophores. Lactophenol cotton blue, 40 ×.

Specimens collected on Day 9 (superficial wound swab), Day 17 (bedside debridement), Day 18 (repeat bedside tissue collection), and Day 19 (operative specimen) all cultured fungal species which were eventually identified as *Mucor* species (Fig. 1). Microscopy was consistent with a *Mucor* species based on non-septated ribbon-like hyphae, sporangia with columella, collarette at base of sporangia, and lack of rhizoids (Fig. 2, Fig. 3, Fig. 4). *Rhizopus* species, the most common cause of mucormycosis, would have rhizoids present. The initial isolate was eventually identified by polymerase chain reaction and sequencing of the internal transcribed spacer region by the national mycology reference laboratory as *Mucor indicus.*

## Discussion

Mucorales are an order of fungi characterized by aseptate hyaline hyphae that have undergone several taxonomic name changes over the years. They are ubiquitous in nature and can be found in food, vegetation, and soil [1]. These species cause a wide spectrum of clinical disease and predominantly affect immunocompromised hosts; the most common risk factors being diabetes mellitus, treatment with glucocorticoids, hematologic malignancies and solid organ transplant [2], [3].

Rhino-orbital-cerebral and pulmonary infections via spore inhalation represent the most common clinical presentations of mucormycosis [4], [5]. Conversely, mucormycosis as a causative agent of post-operative wound infections, particularly in abdominal wounds, is distinctly infrequent and a less described entity. Existing literature on post-operative mucormycosis is predominantly composed of case studies in post-transplant patients, likely attributed to their immunosuppression [6], [7], [8], [9]. Solid organ malignancy, such as bowel cancer without other concomitant immonosuppressive risk factors have not typically been associated with mucormycosis. Surgery itself has been identified as a risk factor for mucormycosis, accounting for 19 % of cases, particularly in immunocompetent hosts [2], [10], [11], [12]. It is worth noting that our patient was not diabetic and had no evidence of pre-morbid immunocompromise prior to his period of critical illness. This underscores the complexity and varied nature of mucormycosis presentations, emphasizing the need for high level of clinical suspicion and nuanced understanding across diverse clinical settings.

In the context of post-operative mucormycosis, the infection’s origin may be iatrogenic, stemming from contaminated surgical wounds. This can occur with contaminated surgical instruments, sutures, or dressings during surgical procedure [13]. Environmental source, including the hospital environment such as hospital linen have been identified as potential source of nosocomial infections and outbreaks related to mucormycosis [13], [14], [15], [16]. In our case, thorough microbiological investigation over the preceding 12 months revealed no other cases of interest, so further environmental investigation was not undertaken.

There are limited case reports describing clinical manifestations such as abdominal wall, chest wall and cutaneous mucormycosis following surgical procedures all highlighting the poor prognosis related to this infection. Nain et al. present a case series of five patients with abdominal wall mucormycosis, all initially diagnosed as necrotizing fasciitis. Among these cases, two were linked to traumatic wounds, while three arose post-surgery. Unfortunately, only two patients survived, with intensive medical therapy and extensive debridement [14]. Similarly, Prasad et al. detailed abdominal wall mucormycosis in an immunocompetent female following epigastric herniorrhaphy. Treatment involved systemic Amphotericin B and repeated surgical debridement, eventually resulting in the removal of most of the anterior abdominal wall and necessitating a split skin graft before the patient made a clinical recovery [17].

Patel et al. reported post-surgical abdominal wound mucormycosis with intestinal involvement in an elderly diabetic man, who did not survive despite multiple debridement and systemic antifungal therapy [15]. Other authors have reported manifestations such as cutaneous mucormycosis following limb amputation [16] and median sternotomy wound mucormycosis infection after cardiothoracic surgery [17]. Table 1 shows key features reported from cases of post-operative mucormycosis in the literature.

**Table 1:** Post operative mucormycosis cases reported in the literature.

Author, year	Site of infection	No of cases	Treatment	Outcome
De Chaumont et al., 2014	Leg	1	Surgery, systemic and local amphotericin B	Survived
Chawla et al., 2007	Sternal wound infection	1	Surgery, liposomal amphotericin B	Died
Abter E et al., 1994	Sternal wound infection	1	Surgery, amphotericin B deoxycholate	Died
Patel et al., 2010	Abdominal wall and intra-abdominal	1	Surgery, amphotericin B deoxycholate	Died
Nain et al., 2015	Abdominal wall	5	Surgery, amphotericin B	3 died, 2 survived
Kumta et al., 2024 (current)	Abdominal wall	1	Limited surgery, liposomal amphotericin B	Died

The optimal management strategies for mucormycosis and the associated patient outcomes have not been extensively studied. There is a scarcity of clinical trials, with evidence largely drawn from case reports and case series [18]. In 2018, the European Confederation of Medical Mycology published global consensus recommendations on the multidisciplinary diagnosis and management of mucormycosis [19]. The consensus strongly advocates for microbiological samples, including direct microscopy and culture of clinical specimens. Early aggressive surgical debridement of the affected area and treatment with liposomal Amphotericin B is recommended. Isavuconazole and posaconazole are used as salvage therapy modalities in case of treatment failure.

All-cause mortality rates for mucormycosis range from 40 % to 80 %, dependent on the underlying conditions and sites of infection [2], [3], [5], [12]. In our case, given the patients age, poor clinical progress and next of kin’s preference to avoid further aggressive measures, extensive debridement was not performed, and the decision was made to transition care to a comfort-based approach.

## Ethical approval

N/a.

## Consent

The patient’s next of kin have provided express informed consent for this case report to be written, published and distributed.

## CRediT authorship contribution statement

**Neha Kumta:** Writing – original draft. **Lawrence Huang:** Investigation, Writing – review & editing. **Gururaj Nagaraj:** Writing – review & editing. **Lindsey Papacostas:** Data curation, Investigation. **Shradha Subedi:** Conceptualization, Investigation, Methodology, Supervision, Validation, Writing – review & editing.

## Declaration of interest statement

The authors report there are no competing interests to declare.
